# Neonatal admission hypothermia and early postnatal growth in a tertiary neonatal unit in Botswana: a prospective observational study

**DOI:** 10.3389/fped.2026.1917615

**Published:** 2026-08-31

**Authors:** Britt Nakstad, Kagiso Mochankana, Jonathan Strysko, Olusegun S. Ewemooje, Odile Sauzet, Kwana Lechiile, Adelaide Lusambili

**Affiliations:** 1 Department of Paediatrics and Adolescent Health, University of Botswana, Gaborone, Botswana; 2 Department of Paediatrics and Adolescent Health, Princess Marina Hospital, Gaborone, Botswana; 3 Botswana-University of Pennsylvania Partnership, Gaborone, Botswana; 4 Children’s Hospital of Philadelphia, Philadelphia, PA, United States; 5 University of Pennsylvania, Philadelphia, PA, United States; 6 Department of Statistics, University of Botswana, Gaborone, Botswana; 7 Bielefeld School of Public Health and Department of Business Administration and Economics, Bielefeld University, Bielefeld, Germany; 8 African International University, Nairobi, Kenya; 9 NextGen for Earth, Nairobi, Kenya

**Keywords:** admission temperature, Botswana, kangaroo mother care, low birth weight, low-resource settings, neonatal hypothermia, postnatal growth, preterm infant

## Abstract

**Introduction:**

Neonatal hypothermia is independently associated with higher morbidity and mortality and remains under-recognized and inconsistently managed in many low-resource neonatal units. Although thermal-care interventions are well established, the relationship between admission temperature and early postnatal growth has been insufficiently described, particularly in sub-Saharan African tertiary neonatal units.

**Methods:**

We conducted a secondary retrospective analysis of a prospective observational study of neonates admitted to a tertiary public neonatal unit in Gaborone, Botswana, from 1 January 2020 to 31 December 2021. Admission hypothermia was defined as a first recorded axillary temperature <36.5°C and classified according to World Health Organization criteria. Growth outcomes included maximal weight loss from birth weight, age at nadir weight, age at regained birth weight, and weekly average weight gain following nadir weight. Multivariable linear regression models assessed associations with admission hypothermia, adjusting for sex, birth weight, gestational age, feeding practices, kangaroo mother care, and disposition.

**Results:**

Among 1,358 neonates, admission hypothermia was common, affecting 66.5% (*n* = 903), with moderate hypothermia accounting for the majority of these cases. The mean (SD) maximal weight loss was 6.75% (5.90%), nadir weight occurred at 4.26 (4.19) days of life, and birth weight was regained at 9.45 (7.61) days of life. Admission hypothermia was not independently associated with these growth outcomes. Gestational age, however, was consistently associated with early growth: older gestational age was associated with lower maximal weight loss, earlier nadir weight, earlier regained birth weight, and shorter time to discharge or transfer among survivors.

**Conclusion:**

In this large prospective cohort from Botswana, admission hypothermia was highly prevalent but was not independently associated with early postnatal growth outcomes. Gestational age was the strongest predictor of early postnatal growth trajectories, highlighting the disproportionate vulnerability of premature infants and the importance of optimizing nutritional and supportive care strategies during early hospitalization.

## Introduction

Thermal protection is a core component of essential newborn care, and the World Health Organization (WHO) defines neonatal hypothermia (HT) as a temperature below 36.5°C, with mild hypothermia or cold stress at 36.0°C–36.4°C, moderate hypothermia at 32.0°C–35.9°C, and severe hypothermia below 32.0°C (1). Low- and middle-income countries (LMICs) continue to carry a disproportionate burden of neonatal mortality and morbidity, and progress toward Sustainable Development Goal target 3.2 remains uneven across sub-Saharan Africa (2, 3). Across low-resource settings, neonatal hypothermia is a common, preventable exposure that compounds the risks related to prematurity, low birth weight (BW), infection, and delayed specialized care (4–8).

Hypothermia during the first minutes, hours, and days after birth is a biologically plausible contributor to poor early growth. Cold stress increases oxygen consumption, glucose use, and metabolic demand, while immature skin, low subcutaneous fat, high surface-area-to-mass ratio, limited vasomotor control, and impaired non-shivering thermogenesis leave preterm and low-birth-weight infants particularly vulnerable (1, 4, 8). Evidence-based prevention therefore relies on a warm delivery environment, prompt drying or occlusive wrapping according to gestational age (GA), warmed transport, continuous temperature monitoring, and skin-to-skin care. Current WHO guidance and recent trial evidence support kangaroo mother care (KMC), including early and prolonged skin-to-skin contact, as an essential intervention for preterm and low-birth-weight infants because it improves survival, reduces infection and hypothermia, supports breastfeeding, and may improve growth (9–13).

Despite high hypothermia prevalence in tropical and semiarid settings, the specific association between neonatal admission temperature and early postnatal growth measures has received little attention. Addressing this knowledge gap is important because early weight loss, timing of lowest weight, time to regain birth weight, and postnatal growth velocity are practical bedside markers of nutrition, hydration, illness severity, and quality of care (14–18). Furthermore, babies who are hypothermic are kept off enteral feeding in many settings, which contributes to a poor growth trajectory. We therefore examined whether admission hypothermia (AT) in a tertiary public neonatal unit in Botswana was associated with early postnatal growth outcomes, while accounting for gestational age, birth weight, feeding practice, KMC, and disposition.

## Methods

### Study population

This prospective observational study collected data prospectively and data were analyzed retrospectively. The study was conducted in a 38-bed neonatal unit of a tertiary public hospital in Gaborone, Botswana. The unit provides care for a broad spectrum of neonatal conditions in a resource-limited environment. Intensive care with invasive ventilation is prioritized for infants with a birth weight ≥900 g or gestational age ≥28 weeks because of high acuity, limited equipment, and staffing constraints. The recruitment period extended from 1 January 2020 to 31 December 2021. Newborns admitted to the neonatal unit were eligible if their mothers or legal guardians received study information and provided written informed consent for collection of clinical data. They were informed that they could withdraw their consent at any time. Clinical data were collected daily for the first 10 days and weekly thereafter until discharge, transfer to another facility, or death. Mothers were continuously updated on their babies’ condition. Approximately 100 newborns per month were admitted to the neonatal ward during the study period and approximately 57% of these neonates were enrolled. Neonates were not recruited during collaborator staff leave days and some weekends. Data were entered into a structured checklist in REDCap and exported to Microsoft Excel for analysis. The study was reported as an observational cohort. We followed the STROBE guidelines for observational studies (https://www.strobe-statement.org/checklists/) and all 22 items were satisfied.

### Variables

The primary exposure variable was the first recorded axillary body temperature at admission to the neonatal unit. Admission hypothermia was defined according to WHO criteria as a temperature <36.5°C and was categorized as mild hypothermia or cold stress (36.0°C–36.4°C), moderate hypothermia (32.0°C–35.9°C), or severe hypothermia (<32.0°C) (1). Normothermia and hyperthermia were also reported. Because the timing of measurement varied by day of life for some infants, the exposure was analyzed as the first recorded admission temperature rather than as a uniformly timed delivery-room or 1-h admission temperature.

Weights were measured using calibrated neonatal scales when available. The prespecified growth outcomes were (1) maximal weight loss from birth weight to nadir weight; (2) day of life of nadir weight; (3) day of life when birth weight was regained; and (4) average weight gain after the nadir weight, expressed as g/kg/day in weekly intervals. Birth weight and gestational age were recorded at admission. Gestational age was primarily estimated using the Ballard score; if this was unavailable, the first day of the last menstrual period was used instead. Additional variables relevant to early growth included sex, day of KMC initiation, daily KMC duration, day of first enteral feeding, day of full enteral feeding, and feeding type. Full feeds were defined as >150 or >140 mL/kg/day when formula fortification was used. Growth outcomes were reported as raw data and we did not use growth chart z-scores.

### Statement of ethics

This project was approved by the institutional review committees of the University of Botswana, the Ministry of Health and Wellness of Botswana, and the Princess Marina Hospital Ethics Committee, all located in Gaborone, Botswana. Written informed consent to participate was obtained from each neonate's parent or legal guardian.

### Sample size

The sample size rationale was based on the expected prevalence of hypothermia from published systematic review evidence (13). Assuming approximately 1,200 annual admissions, a 95% confidence level, and 10% absolute precision, a minimum of 349 neonates was required to estimate hypothermia prevalence. Because 1,358 eligible neonates were enrolled during the 2-year study period, all enrolled participants were included to maximize precision and statistical power for the exploratory regression analyses.

### Statistical analyses

Descriptive statistics are presented as frequencies and percentages for categorical variables and as means with standard deviations for continuous variables. Associations between admission hypothermia and growth outcomes were assessed using linear regression models adjusted for sex, birth weight, gestational age, KMC, feeding type, timing of enteral feeds, timing of full feeds, and end-of-observation status. Regression coefficients are presented with 95% confidence intervals. A two-sided *p*-value <0.05 was considered statistically significant.

## Results

### Population characteristics

A total of 1,358 neonates were included. Overall, 903 infants (66.49%) had hypothermia at admission, and moderate hypothermia (32.0°C–35.9°C) was the predominant category (Table 1). Normothermia was recorded in 314 infants (23.12%), hyperthermia in 16 infants (1.18%), and admission temperature was missing in 125 infants (9.20%).

**Table 1:** Admission temperature categories among neonates admitted to the neonatal unit (*N* = 1,358).

Admission temperature category	*n* (%)
Normothermia (36.5°C–38.0°C)	314 (23.12)
Hyperthermia (>38.0°C)	16 (1.18)
Hypothermia (<36.5°C)	903 (66.49)
Mild (36.0°C–36.4°C)	238 (17.53)
Moderate (32.0°C–35.9°C)	661 (48.67)
Severe (<32.0°C)	4 (0.29)
Missing temperature	125 (9.20)

In the total cohort, the mean (SD) maximal weight loss was 6.75% (5.90%), the lowest weight occurred at 4.26 (4.19) days of life, and birth weight was regained at 9.45 (7.61) days of life. Observed average weight-gain velocities after the lowest weight are presented in Table 2. Data density declined over subsequent weeks because infants were discharged, transferred, died, or lacked complete serial weights. Reported early postnatal growth targets for preterm infants commonly approximate 15–20 g/kg/day, although calculation methods vary and should be interpreted cautiously (17, 18).

**Table 2:** Average growth velocity following nadir weight.

Week after nadir weight	*n*	Mean growth velocity (g/kg/day)	95% CI
1	476	14.18	13.08–15.28
2	341	10.78	9.67–11.89
3	130	13.75	12.22–15.28
4	79	12.15	10.41–13.89
5	39	15.71	12.72–18.70
6	24	13.97	10.39–17.55
7	15	10.88	6.81–14.95
8	8	12.25	7.41–17.09
9	6	12.71	7.92–17.50
10	3	15.33	9.92–20.74

The cohort included neonates with diverse diagnostic profiles (Supplementary Appendix Tables S1 and S2), which probably contributed to variability in growth outcomes. Because many infants had multiple concurrent organ-system diagnoses and because diagnostic data were not structured to support mutually exclusive categories, diagnosis-specific regression analyses were not considered sufficiently reliable.

### Regression analyses

Regression models showed that admission hypothermia was not independently associated with maximal weight loss, day of life of lowest weight, day of life of regained birth weight, or post-lowest-weight average weight gain in the overall cohort after adjustment for prespecified covariates. The regression models showed that the overall population’s hypothermia at admission (AT) was not associated with any of the following growth variables: (1) WL (maximal weight loss from BW to LW); (2) DOL LW (day of life of lowest weight); (3) DOL RBW (regained birth weight); or (4) growth velocity [average weight gain (g/kg/day) in the weeks following DOL LW].

Table 3 presents the regression analysis of factors associated with WL. GA was significantly associated with WL [*β* = −0.43, 95% CI = (−0.56, −0.29); *p* < 0.001]. The negative coefficient suggests that increased gestational age was associated with a reduction in WL. BW showed a negative relationship with WL [*β* = −6.47 × 10⁻⁴; 95% CI = (−13.27 × 10^−4^, 0.31 × 10^−4^); *p* = 0.062], indicating that an increase in birth weight was associated with a slight decrease in WL; however, this association was not statistically significant at the 5% level. Male newborns had lower WL compared with female newborns (*β* = −0.29), although the association was not statistically significant. Similarly, HT was not significantly associated with WL [*β* = 0.09; 95% CI = (−0.70, 0.88); *p* = 0.821], indicating no significant difference in WL between newborns with and without hypothermia.

**Table 3:** Multivariable linear regression analysis of factors associated with maximal weight loss.

Predictor	Adjusted difference in maximal weight loss (%)	95% CI	*p*-Value
Birth weight (per 100 g increase)	−6.47 × 10^−4^	−13.27 × 10^−4^ to 0.31 × 10^−4^	0.062
Gestational age (per week)	−0.43	−0.56 to −0.29	**<0** **.** **001**
Male	−0.29	−0.99 to 0.41	0.416
Admission hypothermia	0.09	−0.70 to 0.88	0.821

Outcome: maximal weight loss from birth weight to nadir weight. *N* = 878. Statistically significant associations are in bold.

The regression analysis of the factors associated with DOL LW showed that GA was significantly associated with DOL LW [*β* = −0.13; 95% CI = (−0.24 to −0.27); *p* = 0.014]. This indicates that an increase in gestational age was associated with an earlier DOL LW. BW showed a non-significant negative relationship with DOL LW (*β* = −2.86 × 10⁻⁴), suggesting that increased birth weight was associated with a slightly earlier DOL LW. Moreover, HT was negatively associated with DOL LW (*β* = −0.27), indicating an earlier DOL LW among newborns with hypothermia compared with those without hypothermia; however, the association was not statistically significant. Male newborns had non-significantly later DOL LW [*β* = 0.33; 95% CI = (−0.23, 0.88); *p* = 0.248] compared with female newborns (see Table 4).

**Table 4:** Multivariable linear regression analysis of factors associated with day of life of lowest weight.

Predictor	Adjusted difference in day of lowest weight (days)	95% CI	*p*-Value
Birth weight (per 100 g increase)	−2.86 × 10^−4^	−8.38 × 10^−4^ to 2.67 × 10^−4^	0.311
Gestational age (per week)	−0.13	−0.24 to −0.03	**0** **.** **014**
Male sex	0.33	−0.23 to 0.88	0.248
Admission hypothermia	−0.27	−0.90 to 0.36	0.405

Outcome: day of life of nadir weight. *N* = 820. Statistically significant associations are in bold.

The regression analysis of factors associated with DOL RBW shows that GA was significantly associated with DOL RBW [*β* = −0.74; 95% CI = (−0.96, −0.51); *p* < 0.001], as shown in Table 5. The negative coefficient suggests that increased gestational age was associated with fewer days required to regain birth weight. BW and HT each showed a negative relationship with DOL RBW (*β* = −5.40 × 10⁻⁴ and −0.76, respectively); however, these associations were not statistically significant. Moreover, male newborns had earlier DOL RBWs compared with female newborns [*β* = −0.63; 95% CI = (−1.8, 0.57); *p* = 0.304], although the association was not statistically significant.

**Table 5:** Multivariable linear regression analysis of factors associated with day of life of regained birth weight.

Predictor	Adjusted difference in day of regained birth weight (days)	95% CI	*p*-Value
Birth weight (per 100 g increase)	−5.40 × 10^−4^	−16.49 × 10^−4^ to 5.70 × 10^−4^	0.340
Gestational age (per week)	−0.74	−0.96 to −0.51	**<0** **.** **001**
Male sex	−0.63	−1.83 to 0.57	0.304
Admission hypothermia	−0.76	−2.15 to 0.63	0.282

Outcome: day of life at regained birth weight. *N* = 483. Statistically significant associations are in bold.

Table 6 shows the regression analysis of factors associated with the day of first enteral feed among the newborns. DOL LW showed a statistically significant positive association with day of first enteral feed [*β* = 0.039; 95% CI = (0.003, 0.076); *p* = 0.034], indicating that a later DOL LW was associated with a later day of first enteral feed. Being male was also significantly associated with day of first enteral feed (*β* = 0.39, *p* = 0.011; 95% CI: 0.089–0.70), suggesting that the male infants had their first enteral feeds later. Furthermore, newborns fed with formula milk had a significantly earlier day of first enteral feed [*β* = −0.47; 95% CI = (−0.82, −0.13); *p* = 0.007] compared with those fed with breast milk. BW and HT were negatively associated with the day of first enteral feed, while GA showed a positive association with the day of first enteral feed; however, these relationships were not statistically significant.

**Table 6:** Multivariable linear regression analysis of factors associated with day of life of first enteral feeding.

Predictor	Adjusted difference in day of first enteral feed (days)	95% CI	*p*-Value
Birth weight (per 100 g increase)	−1.58 × 10^−4^	−4.56 × 10^−4^ to 1.40 × 10^−4^	0.299
Gestational age (per week)	0.02	−0.04 to 0.08	0.522
Male sex	0.39	0.09 to 0.70	**0** **.** **011**
Admission hypothermia	−0.17	−0.51 to 0.18	0.346
Day of lowest weight	0.04	0.00 to 0.08	**0**.**034**
Formula feeding vs. breast milk	−0.47	−0.82 to −0.13	**0**.**007**
Mixed feeding vs. breast milk	−0.21	−0.60 to 0.19	0.306

Outcome: day of life at first enteral feeding. *N* = 719. Statistically significant associations are in bold.

The regression analyses of factors associated with the day of life of first full enteral feed and day of life of starting KMC for less than 3 h per day among the newborns showed that none of the predictor variables were significantly associated with the outcome variables (Tables 7, 8). BW, GA, and HT showed negative associations, while DOL LW and formula milk feeding showed positive associations with DOL of first full enteral feed, as shown in Table 7. BW and HT were negatively associated with KMC duration of less than 3 h/day, while GA was positively associated. The day of starting KMC for more than 3 h [*β* = −0.45; 95% CI = (−0.73, −0.02); *p* = 0.001] was associated with gestational age (Table 9), but this association was not seen for KMC less than 3 h per day (Table 8).

**Table 7:** Multivariable linear regression analysis of factors associated with day of life of full enteral feeding.

Predictor	Adjusted difference in day of full enteral feeding (days)	95% CI	*p*-Value
Birth weight (per 100 g increase)	−7.33 × 10^−4^	−20.44 × 10^−4^ to 5.78 × 10^−4^	0.272
Gestational age (per week)	−0.04	−0.29 to 0.21	0.748
Admission hypothermia	−0.97	−2.45 to 0.51	0.198
Day of lowest weight	0.06	−0.16 to 0.27	0.272
Formula feeding vs. breast milk	0.10	−1.55 to 1.75	0.904
Mixed feeding vs. breast milk	−0.32	−2.39 to 1.74	0.756

Outcome: day of life at full enteral feeding. *N* = 494.

**Table 8:** Multivariable linear regression analysis of factors associated with day of life of initiation of minimal kangaroo mother care (<3 h/day).

Predictor	Adjusted difference in day of KMC initiation (days)	95% CI	*p*-Value
Birth weight (per 100 g increase)	−4.87 × 10^−4^	−10.35 × 10^−4^ to 0.61 × 10^−4^	0.081
Gestational age (per week)	0.02	−0.09 to 0.14	0.669
Male sex	0.27	−0.33 to 0.86	0.376
Admission hypothermia	−0.45	−1.12 to 0.22	0.189

Outcome: day of life at initiation of KMC <3 h/day. *N* = 483.

**Table 9:** Multivariable linear regression analysis of factors associated with day of life of initiation of longer kangaroo mother care (>3 h/day).

Predictor	Adjusted difference in day of KMC initiation (days)	95% CI	*p*-Value
Birth weight (per 100 g increase)	−1.14 × 10^−3^	−2.53 × 10^−3^ to 0.24 × 10^−3^	0.105
Gestational age (per week)	−0.45	−0.73 to −0.18	**0** **.** **001**
Male sex	−0.64	−2.16 to 0.88	0.409
Admission hypothermia	−0.68	−2.54 to 1.17	0.470

Outcome: day of life at initiation of KMC >3 h/day. *N* = 832. Statistically significant associations are in bold.

The regression analysis of factors associated with day of discharge, transfer, or death shows that gestational age GA was significantly associated with day of discharge, transfer, or death [*β* = −0.88; 95% CI = (−1.24, −0.51); *p* < 0.001], as shown in Table 10. This indicates that gestational maturity is a major determinant of postnatal recovery and discharge timing. Furthermore, BW had a significant association with day of discharge, transfer, or death, showing that a higher birth weight was associated with a slightly faster resolution of the postnatal hospital course. The newborns transferred to other facilities left the facility earlier [*β* = −7.54; 95% CI = (−11.71, −3.37); *p* < 0.001] compared to those discharged home, and those who died left even earlier [*β* = −11.60; 95% CI = (−14.41, −8.79); *p* < 0.001], indicating strong assorted discharge status. Note that a transfer is an indication of a high-risk neonatal status associated with a major deviation from standard recovery, while death is an indicator of extreme disease severity in newborns, resulting in the earliest observed day of discharge.

**Table 10:** Multivariable linear regression analysis of factors associated with day of discharge, transfer, or death*.*

Predictor	Adjusted difference in time to end of observation (days)	95% CI	*p*-Value
Birth weight (per 100 g increase)	−2.68 × 10^−3^	−4.42 × 10^−3^ to −0.94 ×10^−3^	**0** **.** **003**
Gestational age (per week)	−0.88	−1.24 to −0.51	**<0**.**001**
Male sex	0.01	−2.00 to 1.98	0.995
Admission hypothermia	0.88	−1.23 to 2.99	0.415
Transferred (vs. discharged home)	−7.54	−11.71 to −3.37	**<0**.**001**
Died (vs. discharged home)	−11.60	−14.41 to −8.79	**<0**.**001**

Outcome: day of discharge, transfer, or death. *N* = 695. Statistically significant associations are in bold.

## Discussion

In this large prospective cohort from a tertiary public neonatal unit in Botswana, admission hypothermia was highly prevalent, affecting over two-thirds of patients. However, no association between admission hypothermia and early growth outcomes was identified within the overall population. In contrast, gestational age was consistently associated with early growth trajectories; older gestational age was associated with lower weight loss, earlier nadir weight, earlier regaining of birthweight, and earlier initiation of enteral feeds. These findings suggest that in this setting, biological maturity and the clinical consequences of prematurity may dominate early growth patterns, consistent with previous studies (4, 7, 13). It also highlights the disproportionate vulnerability of premature infants and the importance of optimizing nutritional and supportive care strategies during early hospitalization.

The four growth outcomes explored in this study are clinically intuitive and feasible in low-resource neonatal units, but they should not be interpreted as interchangeable quality indicators without standardization. Physiological postnatal weight loss reflects contraction of extracellular water, evaporative losses, feeding establishment, illness severity, and fluid management; total body water is highest in the most immature infants (14). Term infants commonly lose up to 7%–10% of birth weight, while preterm infants may lose more depending on maturity, respiratory support, fluid intake, and nutrition strategy (15, 16). The average maximal weight loss of 6.75% and mean regained-birth-weight timing of 9.45 days in this cohort are therefore plausible. However, comparison across studies is limited by inconsistent definitions in published papers and their growth-velocity calculations (17, 18). Our proposed mechanism linking hypothermia to impaired growth is physiologically plausible. This study included all admitted newborns if consent was obtained, and some failed to lose weight, especially the sickest, who may accumulate fluid when ill because their neuroendocrine stress response increases the secretion of antidiuretic hormone, causing the kidneys to retain water. In addition, newborns have immature kidneys with low filtration capacity, and severe illness such as sepsis may cause capillaries to leak, allowing fluid to accumulate in the tissues. Therefore, the absence of an independent association should not be interpreted as evidence that hypothermia is benign. In a related analysis conducted within our neonatal unit, admission hypothermia was independently associated with a 3.5-fold higher risk of death in patients who had hypothermia (3). Likewise, the lack of an observed association between admission hypothermia and growth outcomes does not exclude the possibility that persistent or untreated hypothermia adversely affects feeding and growth. Rather, a single admission temperature may inadequately capture the duration, severity, and cumulative effects of thermal stress experienced during hospitalization. Few studies from sub-Saharan Africa have examined admission temperature and early postnatal growth simultaneously. A recent Ethiopian study reported the time to regain birth weight among preterm neonates but did not evaluate hypothermia as an exposure (19). Other studies have linked regained birth weight and early weight gain to later growth velocity, delayed feeding, or postnatal growth failure (20, 21). Our results add to this literature by showing that, although hypothermia was common, gestational age rather than admission hypothermia was the dominant predictor of the early growth measures available in routine care.

The high prevalence of hypothermia observed in this study is consistent with reports from other low-resource neonatal units. Studies from Nigeria, Uganda, Ethiopia, and Tanzania have shown high hypothermia burdens and have identified prematurity, low birth weight, delayed feeding, suboptimal delivery-room thermal care, and absence of early KMC as important risk factors (13, 22–24). None of our studied growth measures related to admission temperature have been explored thoroughly in papers from sub-Saharan Africa or LMICs, although some document a slightly higher maximal weight loss and duration of hospital stay (25, 26). The prevalence of admission hypothermia in our cohort remained high despite the availability of radiant warmers in labor wards, which reinforces that equipment alone is insufficient. Thermal protection requires coordinated delivery-room preparation, warm transport, early temperature documentation, immediate skin-to-skin care where clinically appropriate, and continuous staff feedback. This study period preceded routine immediate KMC in the labor ward and operating theater at the study site, making the cohort particularly relevant for local quality-improvement planning.

The growth findings should also be interpreted in the context of neonatal nutrition. Further, we report raw data and did not consider using growth chart z-scores. Adequate early protein and energy intake, timely enteral feeding, and appropriate use of the mother's own milk, donor human milk, formula, or fortification are central to preventing postnatal growth failure and supporting neurodevelopment, particularly among very preterm and very-low-birth-weight infants (27–33). In settings where feeding is delayed because of hypothermia or perceived instability, a careful balance is needed: infants should be rewarmed and stabilized promptly, but prolonged avoidable delays in enteral nutrition may worsen cumulative nutritional deficits. This study cannot determine causality between feeding decisions, hypothermia, and growth, but it identifies the following practical targets for improvement: standardized thermal care, early feeding protocols, serial weight-quality assurance, and routine tracking of KMC duration.

This prospective single-center study was conducted in the largest tertiary public neonatal unit in Botswana and included a large real-world cohort of neonates cared for under resource constraints. As the country's principal public referral center, the study population is representative of neonates most dependent on public-sector neonatal services, including infants referred from rural facilities and those with limited access to private care. The study also collected bedside growth, feeding, KMC, and disposition data over 2 years, providing a rare dataset for examining early postnatal growth in an LMIC tertiary neonatal unit.

The study also has important limitations. First, admission temperature was not measured at a standardized postnatal age, and the timing of temperature measurement was not incorporated into the analyses. Consequently, a single admission temperature measurement may not have adequately captured the duration or severity of the thermal stress experienced by individual neonates. Further, a single admission temperature measurement may inadequately reflect cumulative thermal stress or whether clinical interventions mitigated its effects. Second, KMC duration may have been subject to recall bias, incomplete documentation, and misclassification. Third, missing serial weights, intermittent scale availability, staffing shortages, early transfers, deaths, and incomplete postdischarge follow-up reduced the availability of longitudinal growth data and limited assessment of longer-term growth outcomes. The growth analyses may also have been affected by survival bias, as the neonates who died or were transferred early contributed fewer weight measurements than those who remained hospitalized. Fourth, the diagnostic categories overlapped and could not be modeled as mutually exclusive predictors. Finally, residual confounding by illness severity, respiratory support, sepsis, feeding intolerance, maternal illness, delivery-room practices, and transfer pathways likely remained despite multivariable adjustment. These limitations suggest that the absence of an independent association between admission hypothermia and growth outcomes should be interpreted cautiously.

## Conclusions

In this prospective cohort of 1,358 neonates admitted to a tertiary public neonatal unit in Botswana, admission hypothermia was highly prevalent but was not independently associated with early postnatal growth outcomes after adjustment. Gestational age was the strongest and most consistent predictor of maximal weight loss, timing of nadir weight, regaining of birth weight, and disposition timing. These findings suggest that biological maturity exerts a greater influence on early postnatal growth trajectories than a single admission temperature measurement. Future studies should evaluate whether serial measures of thermal instability, illness severity, and nutritional exposures better explain variation in growth among hospitalized neonates, particularly those born preterm.

## Data Availability

The raw data supporting the conclusions of this article will be made available by the authors, without undue reservation.
