# Supplementary material for: Neonatal admission hypothermia and early postnatal growth in a tertiary neonatal unit in Botswana: a prospective observational study
Source: Front Pediatr. 2026 Aug 31;14:1917615. doi: 10.3389/fped.2026.1917615 (PMC13572662; doi:10.3389/fped.2026.1917615)
Supplement: Supplementary file 1 [file Table1.docx]

**Appendices**

## ***Appendix Table 1: Descriptive distribution of neonatal diagnoses by organ-system category. Some neonates have multiple diagnoses.***

| Diagnostic category | Code | n | % of cohort |
| --- | --- | --- | --- |
| Neonatal infections | 1 | 1052 | 75.1 |
| Respiratory disorders | 2 | 721 | 51.5 |
| Cardiovascular disorders | 3 | 110 | 7.9 |
| CNS disorders | 4 | 276 | 19.7 |
| Renal disorders | 5 | 55 | 3.9 |
| Metabolic disorders | 6 | 608 | 43.4 |
| Gastrointestinal disorders | 7 | 100 | 7.1 |
| Haematological disorders | 8 | 78 | 5.6 |
| Trisomies and syndromes | 9 | 28 | 2.0 |
| Observation only | 10 | 97 | 6.9 |

## ***Appendix Table 2: Diagnostic complexity per neonate***

| Number of diagnostic categories | n | % of cohort |
| --- | --- | --- |
| 1 | 400 | 28.6 |
| 2 | 434 | 31.0 |
| 3 | 321 | 22.9 |
| 4 | 127 | 9.1 |
| 5 | 60 | 4.3 |
| 6 | 12 | 0.9 |
| 7 | 2 | 0.1 |
